# A Study on Processing Defects and Parameter Optimization in Abrasive Suspension Jet Cutting of Carbon-Fiber-Reinforced Plastics

**DOI:** 10.3390/ma16227064

**Published:** 2023-11-07

**Authors:** Liucan Li, Nanzhe Xiao, Chuwen Guo, Fengchao Wang

**Affiliations:** School of Low-Carbon Energy and Power Engineering, China University of Mining and Technology, Xuzhou 221116, China; lcli930419@163.com (L.L.); xnz960210@163.com (N.X.); cwguo@cumt.edu.cn (C.G.)

**Keywords:** surface roughness, shoulder damage, kerf taper, carbon-fiber-reinforced plastic, abrasive suspension jet, cutting quality

## Abstract

Abrasive suspension jet (ASJ), an accurate cold-cutting technology, can address traditional processing issues relating to carbon-fiber-reinforced plastics (CFRPs) like tool wear, interlayer delamination, large heat-affected zone, and low surface roughness. This study employed the use of an ASJ to cut CFRPs and an ultra-depth optical microscope to scan the cut surface to analyze interlayer delamination, surface roughness, kerf taper, and shoulder damage. Regression analysis was conducted to establish a prediction model for cutting quality based on surface roughness, kerf taper, and shoulder damage. Various types of CFRP cutting quality were analyzed using jet parameters. It was found that the use of ASJ to process CFRP results in the following defects: The range of surface roughness variation is from 0.112 μm to 0.144 μm. Surface roughness is most influenced by stand-off distance, followed by traverse speed and jet pressure. The range of kerf taper variation is from 4.737° to 10.1°. Kerf taper is most influenced by stand-off distance, followed by jet pressure and traverse speed. The range of shoulder damage variation is from 3.384 μm^2^ to 10 μm^2^. Shoulder damage is most influenced by jet pressure, followed by traverse speed and stand-off distance. A prediction model for cutting quality was developed based on surface roughness, kerf taper, and shoulder damage, providing data support for ASJ cutting of CFRPs. The optimal parameter combination is a stand-off distance of 1 mm, a jet pressure of 30 MPa, and a traverse speed of 30 mm/min.

## 1. Introduction

Carbon-fiber-reinforced plastics (CFRPs) are composite materials made from carbon fibers and resin matrices in various proportions. Compared with traditional metal materials, low density, high strength, corrosion resistance, and weather resistance are among their numerous advantages, and they are widely used in aerospace, nuclear reactors, automobiles, ships, and other fields [1,2]. At present, the processing method for CFRP generally uses mechanical processing methods, which can encounter major issues that are difficult to overcome, such as thermal stress zones, tearing, and lamination at the cut surface [3,4,5,6].

To address these issues, some researchers have turned to laser cutting, ultrasonic machining, water jet machining, and other specialized processing techniques as alternative methods [7,8,9]. Among them, an abrasive water jet (AWJ) is a cold processing technique that is widely used for processing CFRPs due to the absence of a thermal impact zone during the process. In recent years, many researchers have researched the AWJ machining of CFRPs, which avoids the problem of large thermal stress zones. However, AWJ still has shortcomings, such as interlayer delamination, poor surface roughness, and large kerf taper [10,11,12].

To enhance cutting quality, research has been conducted in relation to the aforementioned shortcomings, with some research results as follows. Interlayer delamination is a frequent flaw during AWJ processing. It has been reported in previous studies that enhancing the abrasive mass flow rate can minimize the occurrence of this delamination [13,14]. Some research indicates that modifications to the processing parameters, such as water pressure, stand-off distance, abrasive nozzle diameter, transverse speed, and abrasive flow rate, can also impact delamination [15,16,17,18]. Poor surface roughness is also a common defect during AWJ processing. Research conducted by Schwartzentruber et al. [19] indicates that changes in the process parameters, such as jet pressure, can also impact surface roughness, and selecting an appropriate process parameter can assist in reducing surface roughness. Additionally, a large kerf taper is also a common defect during AWJ processing. Marius et al. [20] compared various processing technologies and discovered that the flexural strength of materials processed via AWJ is superior to that of conventionally milled materials despite AWJ causing greater damage at the material inlet and outlet. Chung et al. [21] demonstrated that the kerf taper angle is related to parameters such as nozzle diameter, traverse speed, and stand-off distance. Youssef H. A. et al. [22] showed that both transverse speed and stand-off distance increases can increase kerf taper.

**Figure 1:**
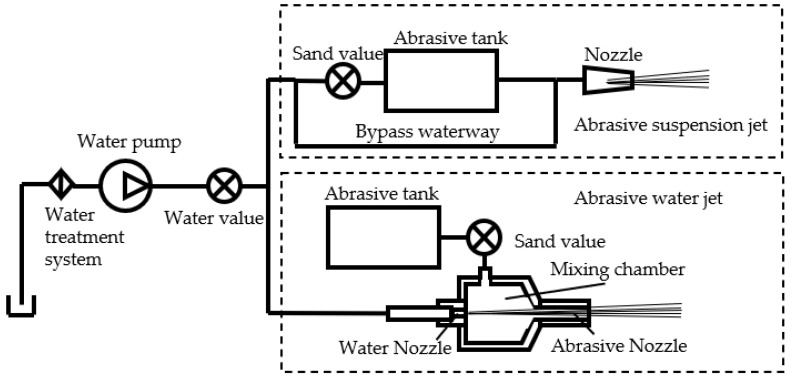
System diagram of abrasive suspension jet and abrasive water jet.

Due to the material properties of CFRPs being highly susceptible to processing through the use of AWJ, it is known that improving the cutting ability of the abrasive while reducing the cutting ability of water can enhance cutting quality under sufficient cutting ability conditions. Compared to AWJ, an abrasive suspension jet (ASJ) exhibits superior jet convergence, lower operating pressure, and higher cutting accuracy. In ASJ, the abrasive is mixed into the jet stream before it forms; an illustrative system diagram is shown in Figure 1. Yang Xinle et al. [23] used a turbulence model in ANSYS FLUENT finite element analysis software (Canonsburg, PA, USA) to simulate the flow field of two types of abrasive jet nozzles and found that the flow field velocity distribution inside the ASJ nozzle is uniform with small turbulence intensity, and the velocity of the abrasive particles at the nozzle outlet is much greater than that of AWJ. Lu Guosheng et al. [24] analyzed the overall force on the abrasive particles in ASJ and compared the pressure required by AWJ and ASJ to reach the same cutting depth through numerical simulation. The results showed that the pressure required by AWJ is about 10 times that of ASJ. Some studies have shown that the mixing method of ASJ is more conducive to accelerating abrasive particles to obtain greater energy. Under the same pressure and power conditions, the processing ability of AWJ jet is only about 20% of ASJ [25].

In recent years, the abrasive suspension jet has been widely used in the processing of various materials. Qiang C.H. et al. [26] studied the influence of process parameters in ASJ technology on the cutting accuracy in stainless steel. Maurya P. et al. [27] conducted a study on the use of ASJ processing for nitrile butadiene rubber. The results showed that ASJ can effectively create grooves on 10 mm thick NBR with high quality. Perec A. et al. [28,29] conducted a study on the influence of ASJ process parameters on the cutting depth of limestone and aluminum. Wang F.C. et al. [30] investigated the influence of ASJ processing parameters on the surface morphology of Ti-6Al-4V(TC4). Ramesha K. et al. [31] studied the effect of ASJ processing parameters on the kerf width and surface roughness of glass-fiber composite materials (GFRPs).

In summary, ASJ shows promise for optimizing cutting quality, but there is currently little research on the processing of CFRPs using ASJ, making it unclear whether it can help to optimize cutting quality. This paper takes this as a starting point and conducts research on the cutting quality and processing defects in ASJ cutting of CFRPs using orthogonal experiments. It compares the processing results of AWJ cutting of CFRPs as a reference, analyzes the defects in ASJ cutting of CFRPs, explores the impact of various processing parameters on cutting quality, and identifies better processing plans, providing guidance for the processing of CFRPs.

## 2. Experimental Section

### 2.1. Experimental Equipment

**Figure 2:**
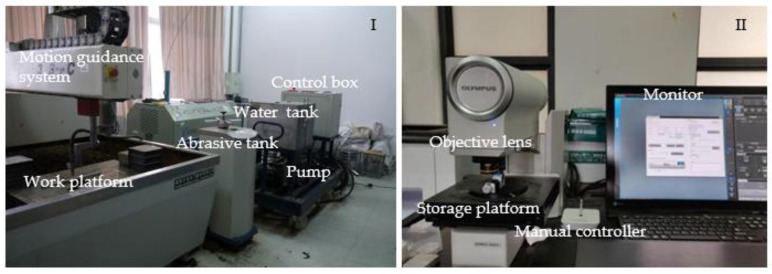
The experimental equipment and data acquisition equipment. (**I**) The system is composed of six parts: a tank, a high-pressure pump, a water pump frequency control box, a motion guidance system, and a work platform. (**II**) The equipment is composed of four parts: an object lens, a storage platform, a manual controller, and a monitor.

The cutting system for ASJ used in this paper is shown in Figure 2I. The execution mechanism adopts the cutting platform produced by Nanjing Dadi Waterjet Company. The adjustable range of the system’s operating pressure ranges from 0 to 35 MPa, the flow control range of the pump ranges from 0 to 16 L/min, and the adjustable range of nozzle traverse speed ranges from 0 to 2500 mm/min.

Figure 2II depicts an OLYMPUS DSX510 ultra-depth optical microscope, which is manufactured by the Olympus Corporation based in Tokyo, Japan. Its zoom ratio is 13.5, and the minimum resolved size is 0.01 μm. This microscope can acquire ultra-depth images or three-dimensional images of cut samples, and multiple observation methods can be used to identify material defects in the samples. The corresponding images can be used to analyze processing defects on the cross-section of ASJ cut CFRP.

### 2.2. Experimental Plan

In this experiment, garnet was used as the abrasive (particle size of 80 mesh, density of 3.6 g/cm^3^, hardness of 7.5 Hm); the target material was a 3K specification CFRP (0/90° layup, resin volume ratio of 33%, carbon fiber content of 175 g/cm^2^, size of 200 mm × 20 mm × 3 mm, and the physical properties of CFRP are shown in Table 1); the nozzle diameter was 0.8 mm; and during processing, the nozzle was perpendicular to the surface of the target material (the impact angle was 0°).

To reduce the experimental workload, this paper adopts the common orthogonal experimental method, and the levels of each factor are shown in Table 2. As a comparison, the experimental parameters for the AWJ used in the experiment were jet pressure of 380 MPa, stand-off distance of 1 mm, and a traverse speed of 200 mm/min. Parameters P, D, and U represent the jet pressure, stand-off distance, and traverse speed of ASJ, respectively. These parameters have a significant impact on the cutting capability of ASJ.

## 3. Analysis and Discussion

For a comprehensive understanding of all the defects involved when cutting CFRPs using ASJ, this section summarizes and analyzes the types and formation reasons of the defects in the experimental results. The advantages and disadvantages of ASJ cutting of CFRPs are comprehensively understood.

**Figure 3:**
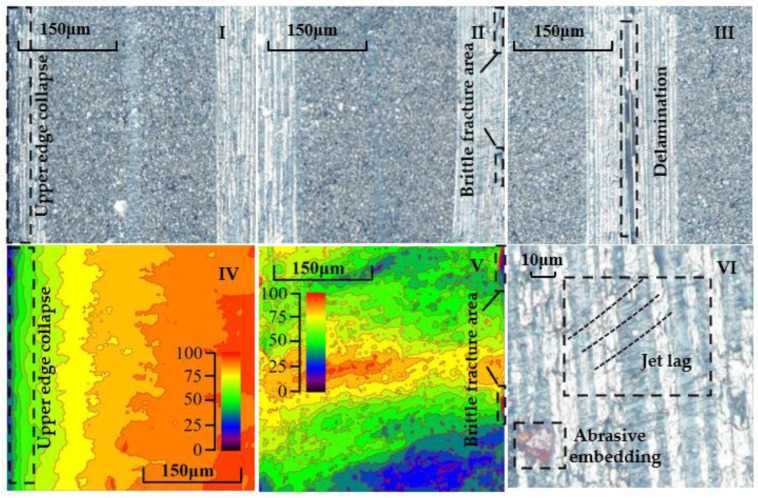
Schematic diagram of section defects. (**I**,**IV**) Defect of shoulder damage. The height of the 40 μm wide area increases rapidly from 20 μm to 70 μm. (**II**,**V**) Defect of brittle fracture. The height of the 20 μm wide area decreases rapidly from 70 μm to 20 μm. (**III**) Defect of delamination with 20 μm width. (**VI**) Jet lag defects and abrasive embedded defects.

Figure 3 shows the defect diagrams of the cutting results in relation to CFRPs when using ASJ, including shoulder damage, brittle fracture, fiber fracture detachment, delamination, abrasive embedding, tailing, etc. Among them, the two diagrams on the left (Figure 3I,IV) show the height contour maps corresponding to the top region of the cutting surface section scanning image, and it can be seen that as the cutting depth increases, the height gradually increases and the contour level is clear. On the left side of them, there is a region with rapid height changes, which is shoulder damage relating to edge collapse. The two diagrams in the middle (Figure 3II,V) show the height contour maps corresponding to the bottom region of the cutting surface section scanning image. Different from the top region, the height curve is undulating, which is related to tailing. In addition, there are some regions that exhibit sudden height reduction on the right side, which is due to the brittle fracture of the CFRP during cutting thickness reduction under jet impact. Figure 3III shows the delamination defect. Figure 3VI shows the tailing and abrasive embedding defects.

### 3.1. Surface Roughness

**Figure 4:**
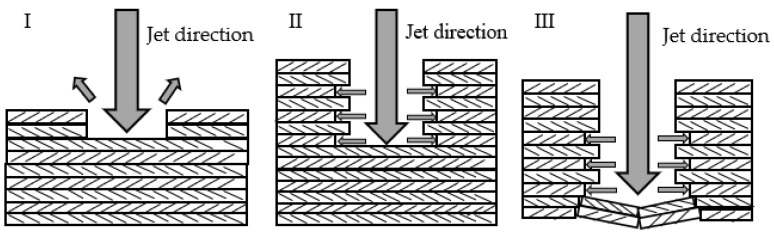
Schematic diagram of impact process. (**I**) The initial stage of impact. The defect of shoulder damage is formed by the erosion of ASJ. (**II**) The development stage of impact. The defect of delamination is formed by the impact of ASJ. (**III**) The final stage of impact. The defect of delamination and brittle fracture is formed by the impact of ASJ.

Surface roughness (Sa) is an important indicator used to evaluate the cutting accuracy of AWJ, and the smaller the Sa, the smoother the surface. As shown in Figure 4I, when the cutting area is located close to the top surface of the material, the jet impinging on the surface reflects mostly outside of the material, causing less impact on the cutting surface. In this region, the kinetic energy of abrasive particles and pure water is high, resulting in strong cutting ability and faster and more thorough grinding of the cutting surface, leading to a smaller Sa in terms of the cut surface. As shown in Figure 4II, when the cutting area is at the middle position of the material, the cutting ability of the jet decreases to some extent. After the jet impacts the surface, it reflects mostly onto the cutting surface, increasing the impact of the jet on the cutting surface. The cutting surface is prone to varying degrees of damage due to the low interlayer shear strength of the material under jet impact, resulting in an increase in Sa on the cutting surface. As Figure 4III indicates, when the cutting area is located near the lower surface of the material, the efficiency of jet cutting is reduced even further, increasing the time required to cut to the same depth and enhancing the impact of the jet on the cutting surface. At the same time, due to the decreased material thickness, the material will sag downward under the impact of the jet. As a brittle material, CFRP experiences very low elongation at breakage. Under the action of AWJ, CFRP will undergo brittle fracture, resulting in further increases in the Sa of the cutting surface. In summary, surface roughness is influenced by parameters such as jet pressure, abrasive flow rate, and traverse speed. Improving the abrasive cutting ability, reducing the water jet cutting ability, and decreasing the traverse speed can all lead to improved cutting quality. Additionally, carbon fiber fracture detachment, abrasive embedding, and tailing phenomenon also affect surface roughness and lower cutting quality.

**Figure 5:**
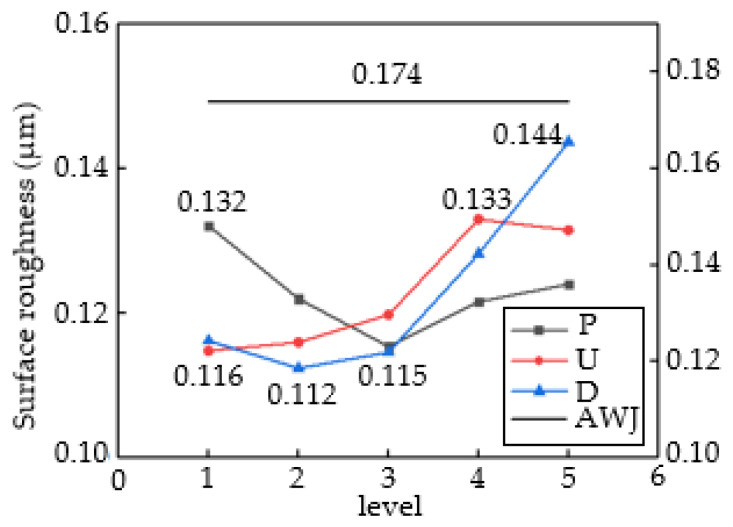
Range analysis of three factors on surface roughness.

Figure 5 shows the analysis results of the range of surface roughness, including the results of the orthogonal experiment (P, U, and D) and control experiment (AWJ). The maximum Sa value in Group D is 0.144μm, corresponding to a stand-off distance of 9 mm; the minimum Sa value is 0.112 μm, corresponding to a stand-off distance of 3 mm. The maximum Sa value in Group P is 0.132 μm, corresponding to a jet pressure of 10 MPa, and the minimum Sa value is 0.115 μm, corresponding to a jet pressure of 20 MPa. The maximum Sa value in Group U is 0.133 μm, which corresponds to a traverse speed of 120 mm/min; the minimum Sa value is 0.115 μm, corresponding to a traverse speed of 30 mm/min.

It can be inferred that the optimal parameter combination consists of a stand-off distance of 3 mm, a jet pressure of 20 MPa, and a traverse speed of 30 mm/min. Comparing the range values, it can be seen that the influence of jet parameters on Sa, in descending order, is D > U > P. The AWJ line in the graph represents control group data. Compared to all four sets of data, it can be seen that the cutting quality of ASJ cutting is better. This is because the jet pressure of AWJ is much greater than that of ASJ, and its value can be calculated using the formula for the maximum compressive stress normal to the surface of the workpiece during the impact of abrasive particles and water jet on CFRP mentioned in [32].

(1) $$\sigma_{p\max}=0.83\rho_{1}^{1/5}{\left(\frac{1-\mu_{1}^{2}}{\pi E_{1}}+\frac{1-\mu_{2}^{2}}{\pi E_{2}}\right)}^{-4/5}P_{0}^{1/5}{\left(\frac{1-\omega_{1}}{1000}+\frac{\omega_{1}}{\rho_{1}}\right)}^{1/5}$$

(2) $$\sigma_{w\max}=2P_{0}(1-\omega_{1})$$

In which *P*_0_ is the jet outlet pressure; *μ*_1_, *E*_1_, *ρ*_1_, and *ω*_1_ are the Poisson’s ratio, Young’s modulus, density, and mass fraction of the abrasive particles, respectively; and *μ*_2_ and *E*_2_ are the Poisson’s ratio and Young’s modulus of the CFRP, respectively.

By calculating according to Equation (2) and comparing with the values in Table 1, a pure water jet directly impacting the surface of the material can process CFRP, and a reflecting jet at a certain angle can also directly damage the carbon fiber material, which easily leads to the damage and delamination shown in Figure 4III. At the same time, in the part of the material near the ground, the area of brittle material failure increases, greatly reducing the cutting quality. In summary, in terms of surface roughness, selecting ASJ is more conducive to reducing surface roughness and improving cutting quality.

Table 3 presents the variance analysis results in terms of Sa. The F-value results show that these three factors have a certain influence on the surface roughness during the water jet processing of CFRP laminates. The D parameter has a significant impact on Sa, and the U parameter also has a significant impact, but its influence is weaker than the former. The P parameter does not have a significant impact. Based on the range analysis, in the processing parameters of ASJ processing of CFRP laminates, the stand-off distance and traverse rate are important parameters. To obtain a better cutting quality, both parameters need to be at a lower numerical level. The selection range of pressure parameters is relatively broad as long as it can ensure penetration of the material.

### 3.2. Kerf Taper

**Figure 6:**
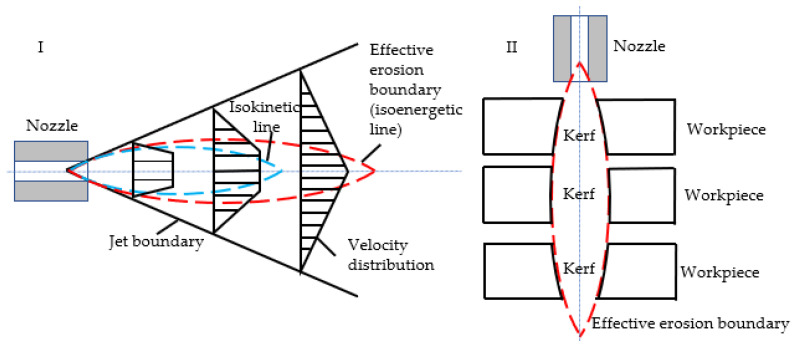
Schematic diagram of jet structure and slit shape. (**I**) The red line is the critical energy contour of the jet, which is the contour line of the slit in the ideal state. (**II**) Under the same processing parameters, the material will exhibit 3 different profiles depending on the distance from the nozzle.

Kerf taper is an important metric for assessing the cutting accuracy of ASJ. It is defined as the difference between the width of the upper surface and that of the lower surface of the ASJ cutting seam divided by two. As the depth of the cutting seam increases, the energy of the ASJ decays, reducing the ability of the ASJ to remove material, coupled with changes in the velocity of the ASJ, ultimately leading to the formation of the kerf taper. Changing the cutting ability of the ASJ, such as by altering the jet pressure, traverse speed, impact angle, abrasive particle size, abrasive flow rate, and abrasive hardness parameters, will alter the kerf taper. Based on the flow field structure of the water jet (Figure 6I), the jet pressure and stand-off distance parameters can modify the effective erosion boundary of the jet and the contact location with the workpiece, which alters the contour and taper of the cut, as shown in Figure 6II.

**Figure 7:**
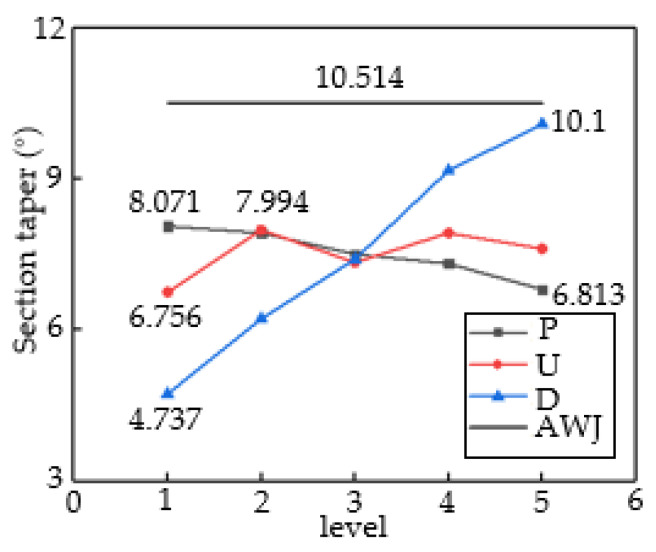
Range analysis of three factors for section taper.

Figure 7 presents the results of the analysis of the range of kerf taper (γ). The stand-off distance and impact angle show a linear positive correlation, and as the D increases, the γ value also increases. The maximum γ value in the D group is 10.1°, corresponding to a stand-off distance of 9 mm, and the minimum γ value is 4.737°, corresponding to a stand-off distance of 1 mm, with a range difference of 5.35°. The maximum γ value in the P group is 8.071°, corresponding to a jet pressure of 10 MPa, and the minimum γ value is 6.813°, corresponding to a jet pressure of 30 MPa, with a range difference of 1.258°. The maximum γ value in the U group is 7.994°, corresponding to a traverse speed of 60 mm/min, and the minimum γ value is 6.756°, corresponding to a traverse speed of 30 mm/min, with a range difference of 1.238°. A stand-off distance of 1 mm, jet pressure of 30 MPa, and traverse speed of 30 mm/min yield the best-processed surface. The influence of each factor on kerf taper from largest to smallest is D > P > U. The AWJ line in the graph represents control group data. Comparing the four sets of data, it can be seen that the cutting quality of the ASJ is better. This is because the jet pressure of AWJ is much greater than that of ASJ. According to Formula (2), a pure water jet can also process CFRP under conditions where the processing ability of the abrasive particles is equal. Therefore, under conditions where the total processing ability of AWJ is higher than that of ASJ, its sample kerf taper angle is smaller in accordance with expectations, indicating that AWJ jet is superior to ASJ in terms of kerf taper evaluation criteria.

Table 4 presents the results of the analysis of variance for kerf taper. Based on the F-value data, it can be inferred that all three factors have a significant impact on kerf taper during ASJ processing of CFRP laminates. Stand-off distance has a significant impact on kerf taper, while both traverse speed and jet pressure parameters have no significant impact. To achieve a better cutting quality, it is necessary to select higher pressure, lower stand-off distance, and traverse speed.

### 3.3. Shoulder Damage

**Figure 8:**
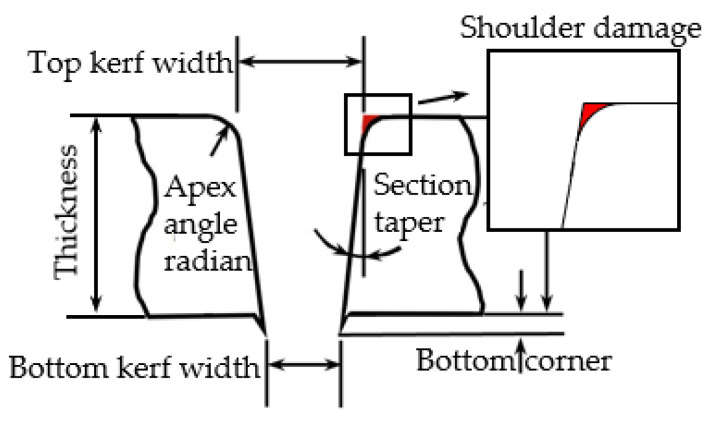
Defect diagram.

Shoulder damage (SD) refers to the formation of an arcuate corner at the top edge of the kerf during ASJ processing of materials, as shown in Figure 8. SD is an important indicator that measures the cutting accuracy of ASJ, with its value representing the area enclosed by the intersection of the cut surface along the taper angle with the top surface and the actual cutting surface, manifested as a rapid change in scanning height in the top region of the cut surface, as shown in Figure 3IV within the black dashed box. As CFRP is relatively easy to process, SD mainly depends on the cutting ability of ASJ. If the cutting speed is slow, the SD will be larger. Therefore, the severity of shoulder damage is related to parameters such as jet pressure, stand-off distance, and traverse speed.

**Figure 9:**
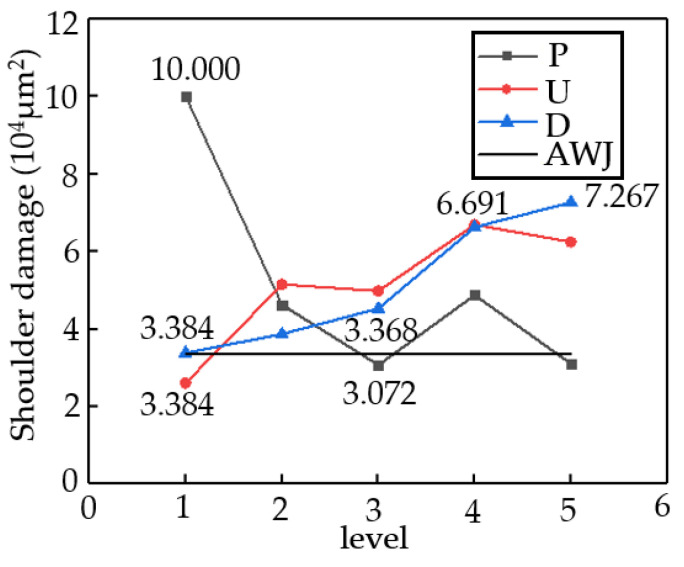
Range analysis of three factors for shoulder damage.

Figure 9 presents a range analysis of the shoulder damage (SD). The smaller the value of SD, the smaller the damaged area and the better the cutting quality. In Group D, the maximum SD value is 72,668 μm^2^ with a corresponding D of 9 mm, while the minimum SD value is 33,840 μm^2^ with a corresponding D of 1 mm, with a range value of 38,828 μm^2^. In Group P, the maximum SD value is 99,992 μm^2^ with a corresponding P of 10 MPa, while the minimum SD value is 30,719 μm^2^ with a corresponding P of 20 MPa, with a range value of 69,273 μm^2^. In Group U, the maximum SD value is 46,256 μm^2^ with a corresponding U of 60 mm/min, while the minimum SD value is 26,069 μm^2^ with a corresponding U of 30 mm/min, with a range value of 20,187 μm^2^. Therefore, the optimal processing quality can be obtained when the jet parameters are D = 1 mm, P = 20 MPa, and U = 30 mm/min. Based on the range values, the jet parameters affecting SD from largest to smallest are P > U > D. The common feature of SD being large at several points is that their cutting abilities are low, and either P is small (10 or 15 MPa), U is high (120 or 160 mm/min), or both. When the cutting ability exceeds a certain value, the decrease in SD slows down. Comparing the two cutting methods, when the abrasive cutting abilities of the two jet modes are similar, the processing ability of AWJ pure water jet is much greater than that of ASJ pure water jet, resulting in less shoulder damage during processing, consistent with the trend shown in Figure 9. In summary, AWJ performs better in terms of the shoulder damage index.

Table 5 presents the variance analysis results of the shoulder damage. Based on the F-value results, all three factors have a certain impact on the shoulder damage of AWJ processing of CFRPs. The jet pressure parameter has a significant impact on the shoulder damage area, while the stand-off distance parameter and the traverse speed parameter of the cutting head have no significant impact on the injury area. Combining the range analysis, the jet pressure parameter can be selected at a relatively large value level, while the stand-off distance and traverse speed parameters are preferably at a lower value level.

### 3.4. Interlayer Delamination

**Figure 10:**
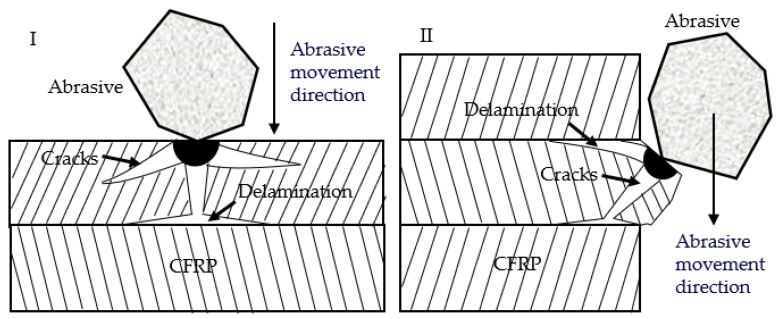
Schematic diagram of the generation of delamination defects. (**I**) The delamination produced by abrasive particles impacting the plane. (**II**) The delamination produced by abrasive particles impacting the protrusion on the cutting surface.

Interlayer delamination is one of the common defects observed during the AWJ cutting of CFRPs, mainly manifesting as gaps between different layers of carbon fiber board, which can affect overall performance. The formation of delamination is divided into internal and external causes. The former refers to the defects formed during the manufacturing process due to an uneven distribution of materials and other reasons, while the latter refers to the formation of layering under the combined action of abrasive particles and the water jet. As shown in Figure 10I, particles impact the surface of the material, causing cracks on the surface, and then water seeps into the cracks through water wedge action, allowing the cracks to expand and extend to the layer connection, where they cause interlayer delamination by squeezing and breaking the layer boundary. As shown in Figure 10II, particles impact the convex part of the cutting surface, causing cracks at the convex part, and then under the impact of water, the convex part of the material undergoes brittle fracture and forms irregular pits with cracks at the cutting surface. Subsequently, under the action of a water wedge, the crack extends to the layer boundary and forms interlayer delamination. In summary, the main cause of interlayer delamination is the water wedge action of a pure water jet. Therefore, jet pressure is the main factor affecting interlayer delamination, and compared with AWJ, ASJ has lower jet pressure and can be expected to have fewer layering defects.

**Figure 11:**
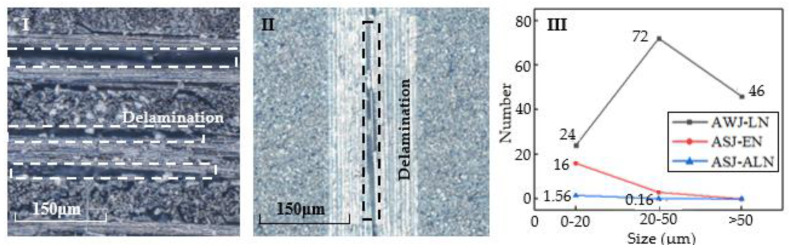
Schematic diagram and quantity statistical diagram of delamination defects. (**I**) Delamination defect of the control group during cutting using AWJ. (**II**) Delamination defect of the experimental group during cutting using ASJ. (**III**) Comparison chart of delamination defects between AWJ and ASJ.

Figure 11I shows the cross-section scan when using AWJ. As can be seen from the figure, there are a large number and size of layering defects. Figure 11II shows the cross-section scan when using ASJ. As can be seen from the figure, there are no multiple layering defects, and the size is relatively small. The specific comparison of quantity and size is shown in Figure 11III. AWJ-LN represents the number of layers of different sizes when using AWJ. There are a total of 142 layers, distributed as follows: 24 layers are 0–20 μm, mainly present at the top of the cross-section; 72 layers are 20–50 μm, mainly present in the middle and lower part of the cross-section; and 46 layers are greater than 50 μm, mainly present at the bottom of the cross-section. ASJ-EN represents the number of experiments in which corresponding size layering defects exist when using ASJ, i.e., there are 16 groups with 0–20 μm layering defects in 25 groups of experiments, 3 groups with 20–50 μm layering defects, and no greater than 50 μm layering defects. ASJ-ALN represents the average number of layers of different sizes in the results of 25 groups of ASJ experiments, of which there are 1.56 layers between 0 and 20 μm, 0.16 layers between 20 and 50 μm, and no layers greater than 50 μm. Comparing the data, it can be seen that using ASJ can significantly reduce layering defects and improve cutting quality, and the comparisons are in line with expectations.

### 3.5. Reference Comparison

A comparative analysis was conducted with existing research results to ensure the accuracy of the experimental results.

Yang Jingxu et al. [33] used AWJ to cut CFRP. A total of 17 groups of experiments were conducted around jet pressure, stand-off distance, and traverse speed, and the surface roughness and kerf taper of the section were analyzed. The range of surface roughness is between 4.73 μm and 6.33 μm, which is an order of magnitude higher than the surface roughness results cut by ASJ; the range of kerf taper is between 3.29° and 6.42°, which is smaller than the kerf taper results cut by ASJ. Both are consistent with the conclusions drawn in this paper.

## 4. Optimization

This section aims to further investigate the impact of ASJ processing parameters on the cutting quality of CFRP materials and select the optimal parameter combination. A quantitative analysis will be conducted to examine the influence of processing parameters on cutting quality. Using the linear regression method, prediction models will be established for surface roughness, kerf taper, edge damage, and overall cutting quality based on the three factors.

### 4.1. Model Building

This section proposes the following jet-cutting quality prediction model, using jet pressure, stand-off distance, and lateral speed as independent variables and surface roughness, cutting surface taper, upper edge collapse damage, and jet-cutting quality based on the three factors as dependent variables.

(3) $$H=KD^{\left(c_{1}+c_{4}L_{n}D\right)}P^{\left(c_{2}+c_{5}L_{n}P\right)}U^{\left(c_{3}+c_{6}L_{n}U\right)}$$

In which *H* is the prediction parameter, which can represent the surface roughness (Sa), the cutting surface taper (γ), the upper edge shoulder damage (SD), the jet-cutting quality based on surface roughness *I_Sa_*, or the jet-cutting quality based on kerf taper *I γ* or based on the jet-cutting quality *I_SD_* of the upper edge shoulder injury; *K* is a constant coefficient related to the physical properties of CFRP; *P* is jet pressure; *U* is the lateral velocity; *D* is the stand-off distance; and *c*_1_–*c*_6_ are undetermined coefficients.

By manipulating Equation (3), we obtain the following:

(4) $$y=c_{0}+c_{1}x_{1}+c_{2}x_{2}+c_{3}x_{3}+c_{4}x_{1}^{2}+c_{5}x_{2}^{2}+c_{6}x_{3}^{2}$$

In which *y* = *L_n_H*; *c*_0_ = L_n_k; *x*_1_ = *L_n_D*; x_2_ = *L_n_P*; and *x*_3_ = *L_n_U*.

By using the least squares method and combining the raw experimental data, we can obtain the seven unknown coefficients shown in Equation (4) and then substitute them into Equation (3) to obtain the prediction models for each parameter.

### 4.2. Model Solution

Based on the linear regression method for establishing prediction models described above, the values of the experimental conditions and experimental results from 25 sets of experiments were taken as logarithms and then subjected to linear regression calculations to obtain the coefficients to be determined; the results are shown in Table 6.

Based on the results of regression analysis, the following regression equations for the cutting quality of CFRP via ASJ cutting can be obtained:

(5) $$y=57.5+1.595x_{1}-7.86x_{2}+0.724x_{3}+0.13x_{1}^{2}+0.234x_{2}^{2}+0.0465x_{3}^{2}$$

(6) $$y=-49.6+1.511x_{1}+6.29x_{2}-1.8x_{3}+0.0994x_{1}^{2}-0.194x_{2}^{2}-0.1378x_{3}^{2}$$

(7) $$y=183+3.94x_{1}-24.6x_{2}-8.25x_{3}+0.295x_{1}^{2}+0.705x_{2}^{2}-0.641x_{3}^{2}$$

The *p*-values of linear regression for Formulas (5)–(7) are 0, 0, and 0.001, indicating that the reliability of the three regression equations is close to 100%. Therefore, the prediction models for the cutting quality of CFRPs via ASJ cutting are, respectively, the following:

(8) $$I_{Sa}=9.37E^{24}D^{\left(1.595+0.13L_{n}D\right)}P^{\left(-7.86+0.234L_{n}P\right)}U^{\left(0.724+0.0465L_{n}U\right)}$$

(9) $$I_{\gamma }=2.88E^{-22}D^{\left(1.511+0.0994L_{n}D\right)}P^{\left(6.29-0.194L_{n}P\right)}U^{\left(-1.8-0.1378L_{n}U\right)}$$

(10) $$I_{SD}=2.99E^{79}D^{\left(3.94+0.295L_{n}D\right)}P^{\left(-24.6+0.705L_{n}P\right)}U^{\left(-8.25-0.641L_{n}U\right)}$$

After the above linear regression calculation, a prediction model for the cutting quality, surface roughness, kerf taper, and shoulder damage in ASJ cutting of CFRPs was obtained, providing data support for ASJ cutting of CFRPs and guiding the selection of optimal impact parameters. After calculation, the optimal parameter combination is a stand-off distance of 1 mm, a jet pressure of 30 MPa, and a traverse speed of 30 mm/min.

## 5. Conclusions

This paper conducted experimental research on the cutting quality and process optimization in ASJ cutting of CFRPs. The main conclusions are as follows:

(1) When it comes to the evaluation criterion of surface roughness, selecting ASJ is more conducive to improving the cutting quality. The factors that impact surface roughness include stand-off distance, traverse speed, and jet pressure, with stand-off distance having the greatest impact, followed by traverse speed and jet pressure. The optimal parameter combination is a stand-off distance of 3 mm, a jet pressure of 20 MPa, and a traverse speed of 30 mm/min.
(2) Regarding the evaluation criterion of kerf taper, selecting AWJ is more beneficial to improving cutting quality. The factors that affect the taper of the cutting surface are stand-off distance, jet pressure, and traverse speed, in descending order of importance. The optimal parameter combination is a stand-off distance of 1 mm, a jet pressure of 30 MPa, and a traverse speed of 30 mm/min.
(3) In terms of the evaluation criterion of shoulder damage, the effect of AWJ is more conducive to improving the cutting quality. The factors that have the greatest impact on shoulder damage are jet pressure, traverse speed, and stand-off distance, in that order. The optimal parameter combination is a stand-off distance of 1 mm, a jet pressure of 20 MPa, and a traverse speed of 30 mm/min.
(4) In terms of the evaluation criterion of delamination defects, selecting ASJ is more conducive to improving the cutting quality.
(5) Using linear regression methods, a prediction model for surface roughness, kerf taper, and shoulder damage, as well as jet-cutting quality based on the three factors, was established to provide data support for the ASJ cutting of CFRP, guiding the selection of optimal impact parameters. The optimal parameter combination is a stand-off distance of 1 mm, jet pressure of 30 MPa, and traverse speed of 30 mm/min.

## Figures and Tables

**Table 1:** CFRP physical properties.

Factor	Value
Young’s modulus (GPa)	181
Shear strength (MPa)	105
Transverse tensile strength (MPa)	1700
Longitudinal compressive strength (MPa)	81.3
Transverse compressive strength (MPa)	1200
Longitudinal compressive strength (MPa)	40
Poisson’s ratio	0.31

**Table 2:** Experimental design.

Factor	Levels
P (MPa)	10, 15, 20, 25, 30
U (mm/min)	30, 60, 80, 120, 160
D (mm)	1, 3, 5, 7, 9

**Table 3:** Calculation results of surface roughness variance.

	Parameter	SST	DF	MS	F	P
Sa (µm)	D	0.003	4	0.001	8.337	0.002
	P	0.001	4	0.000	1.748	0.204
	U	0.001	4	0.000	3.639	0.037

**Table 4:** Calculation results of inclination angle variance.

	Parameter	SST	DF	MS	F	P
γ (°)	D	94.099	4	23.525	42.657	0.001
	P	5.058	4	1.264	2.293	0.119
	U	5.083	4	1.271	2.304	0.118

**Table 5:** Calculation results of shoulder damage variance.

	Parameter	SST	DF	MS	F	P
SD (µm^2^)	D	5.897 × 10^9^	4	1.474 × 10^9^	2.314	0.117
	P	1.619 × 10^10^	4	4.047 × 10^9^	6.353	0.006
	U	5.034 × 10^9^	4	1.258 × 10^9^	1.976	0.163

**Table 6:** Calculation results of linear regression.

Coefficient	Sa		γ		SD	
	Numerical Solution	Confidence Interval	Numerical Solution	Confidence Interval	Numerical Solution	Confidence Interval
$c_{0}$	57.5	[−7.18, 122.18]	−49.6	[−149.4, 50.2]	183	[–260, 626]
$c_{1}$	1.595	[0.89, 2.30]	1.511	[0.43, 2.6]	3.94	[−0.88, 8.76]
$c_{2}$	−7.86	[−15.6, −0.12]	6.29	[−5.67, 18.25]	−24.6	[−77.72, 28.52]
$c_{3}$	0.724	[−0.58, 2.03]	−1.8	[−3.82, 0.22]	−8.25	[−17.17, 0.67]
$c_{4}$	0.13	[0.07, 0.19]	0.0994	[0.0065, 1.92]	0.295	[0.54, 1.36]
$c_{5}$	0.234	[−0.01, 0.47]	−0.194	[−0.55, 0.16]	0.705	[−0.89, 2.3]
$c_{6}$	0.0465	[−0.05, 0.14]	−0.138	[−0.29, 0.011]	−0.641	[−1.3, 0.018]
	R^2^ = 66.56%, P = 0, F = 8.96		R^2^ = 86.39%, P = 0, F = 26.38		R^2^ = 60.31%, P = 0.001, F = 7.08	

## Data Availability

Data is available on request from the corresponding author.
